# Diclomezine: 6-(3,5-dichloro-4-methyl­phen­yl)pyridazin-3(2*H*)-one

**DOI:** 10.1107/S1600536810047409

**Published:** 2010-11-20

**Authors:** Hyunjee Kim, Tae Ho Kim, Jineun Kim, Ki-Min Park

**Affiliations:** a Department of Chemistry and Research Institute of Natural Sciences, Gyeongsang National University, Jinju 660-701, Republic of Korea

## Abstract

In the title compound, C_11_H_8_Cl_2_N_2_O, the benzene and pyridazine rings are tilted by 8.6 (1)° relative to each other. In the crystal, pairs of inter­molecular N—H⋯O hydrogen bonds form centrosymmetric dimers. π–π contacts with centroid–centroid distances of 3.698 (2) and 3.751 (1) Å and halogen–halogen inter­actions [3.379 (1) Å] also stabilize the structure.

## Related literature

For information on the toxicity and fungicidal properties of the title compound, see: Sankyo (1998 ▶). For a related structure, see: Prout *et al.* (1994 ▶).
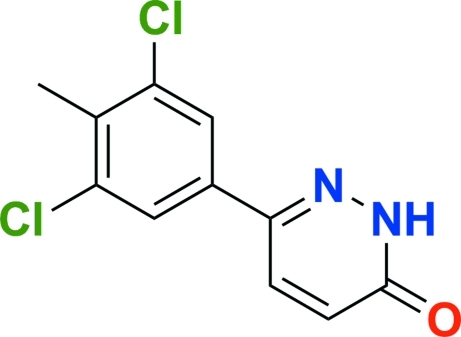


## Experimental

### 

#### Crystal data

C_11_H_8_Cl_2_N_2_O
*M* *_r_* = 255.09
Monoclinic, 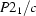

*a* = 9.745 (4) Å
*b* = 13.850 (5) Å
*c* = 8.481 (3) Å
β = 111.557 (6)°
*V* = 1064.7 (7) Å^3^
*Z* = 4
Mo *K*α radiation
μ = 0.59 mm^−1^
*T* = 173 K
0.19 × 0.09 × 0.08 mm

#### Data collection

Bruker APEXII CCD diffractometer
Absorption correction: multi-scan (*SADABS*; Sheldrick, 1996 ▶) *T*_min_ = 0.897, *T*_max_ = 0.955
10410 measured reflections
2657 independent reflections
2038 reflections with *I* > 2σ(*I*)
*R*_int_ = 0.042

#### Refinement

*R*[*F* ^2^ > 2σ(*F* ^2^)] = 0.034
*wR*(*F* ^2^) = 0.091
*S* = 1.07
2657 reflections
146 parameters
H-atom parameters constrained
Δρ_max_ = 0.30 e Å^−3^
Δρ_min_ = −0.26 e Å^−3^

### 

Data collection: *APEX2* (Bruker, 2006 ▶); cell refinement: *SAINT* (Bruker, 2006 ▶); data reduction: *SAINT*; program(s) used to solve structure: *SHELXS97* (Sheldrick, 2008 ▶); program(s) used to refine structure: *SHELXL97* (Sheldrick, 2008 ▶); molecular graphics: *SHELXTL* (Sheldrick, 2008 ▶); software used to prepare material for publication: *SHELXTL* and *DIAMOND* (Brandenburg, 1998 ▶).

## Supplementary Material

Crystal structure: contains datablocks global, I. DOI: 10.1107/S1600536810047409/sj5054sup1.cif

Structure factors: contains datablocks I. DOI: 10.1107/S1600536810047409/sj5054Isup2.hkl

Additional supplementary materials:  crystallographic information; 3D view; checkCIF report

## Figures and Tables

**Table 1:** Hydrogen-bond geometry (Å, °)

*D*—H⋯*A*	*D*—H	H⋯*A*	*D*⋯*A*	*D*—H⋯*A*
N1—H1⋯O1^i^	0.88	1.90	2.771 (2)	172

Symmetry code: (i) 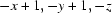.

**Fig. 1.**
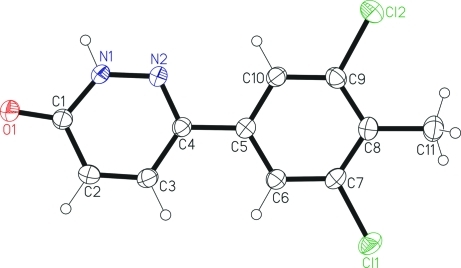
The molecular structure of the title compound, showing displacement ellipsoids drawn at the 50% probability level.

**Fig. 2.**
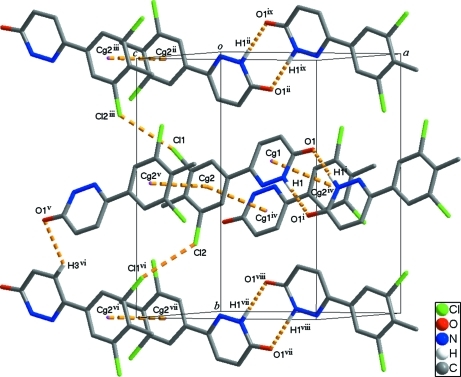
Crystal packing of the title compound with intermolecular N—H···O hydrogen bonds, π—π and halogen—halogen interactions shown as dashed lines. H atoms not involved in intermolecular interactions have been omitted for clarity. Cg1 and Cg2 are the centroids of the phenyl and pyridazine rings, respectively. (Symmetry codes: i) 1 - x, 1 - y, -z; ii) x, 0.5 - y, 1/2 + z; iii) -x, -1/2 + y, 1.5 - z; iv) 1 - x, 1 - y, 1 - z; v) -x, 1 - y, 1 - z; vi) -x, 1/2 + y, 1.5 - z; vii) x, 1.5 - y, 1/2 + z; viii) 1 - x, 1/2 + y, 0.5 - z; ix) 1 - x, -1/2 + y, 0.5 - z) .

## supplementary crystallographic information

### Comment

Declomezine (systematic name: 6-(3,5-dichloro-4-methylphenyl)- 3(*2H*)-pyridazinone), is a well known fungicide (Sankyo 1998). In this paper, the structure of the title pyridazinone derivative is reported.

In the molecular structure of the title compound (Scheme 1, Fig. 1), the dihedral angle between the phenyl ring and the pyridazine ring is 8.6 (1) °, compared to the value of 18.0 ° in the similar compound 6-phenyl-3(*2H*)-pyridazinone (Prout *et al.*, 1994). Bond lengths and angles observed here are also similar to those in 6-phenyl-3(*2H*)-pyridazinone.

In the crystal structure, as shown in Fig. 2, there are pair-wise intermolecular N—H···O hydrogen bonds (Table 1; symmetry code as in Fig. 2). Weak intermolecular π—π and halogen···halogen interactions also exist [*Cg*1···*Cg*2^iv^ 3.75 Å, *Cg*2···*Cg*2^v^ 3.70 Å and Cl1···Cl2^iii^ 3.379 (1) Å; *Cg*1 and *Cg*2 are the centroids of the phenyl and pyridazine rings, respectively.]. These intermolecular interactions contribute to the stabilization of the packing.

### Experimental

The title compound was purchased from the Dr. Ehrenstorfer GmbH Company. Slow evaporation of a solution in CH_2_Cl_2_ gave single crystals suitable for X-ray analysis.

### Refinement

All H-atoms were positioned geometrically and refined using a riding model with d(N—H) = 0.88 Å, *U*_iso_ = 1.2*U*_eq_(N) for pyrazine, d(C—H) = 0.95 Å, *U*_iso_ = 1.2*U*_eq_(C) for aromatic, d(C—H) = 0.98 Å, *U*_iso_ = 1.5*U*_eq_(C) for methyl protons.

### Crystal data

**Table:**

C_11_H_8_Cl_2_N_2_O	*F*(000) = 520
*M**_r_* = 255.09	*D*_x_ = 1.591 Mg m^−^^3^
Monoclinic, *P*2_1_/*c*	Mo *K*α radiation, λ = 0.71073 Å
Hall symbol: -P 2ybc	Cell parameters from 3791 reflections
*a* = 9.745 (4) Å	θ = 2.9–28.2°
*b* = 13.850 (5) Å	µ = 0.59 mm^−^^1^
*c* = 8.481 (3) Å	*T* = 173 K
β = 111.557 (6)°	Block, colourless
*V* = 1064.7 (7) Å^3^	0.19 × 0.09 × 0.08 mm
*Z* = 4	

### Data collection

**Table:**

Bruker APEXII CCD diffractometer	2657 independent reflections
Radiation source: fine-focus sealed tube	2038 reflections with *I* > 2σ(*I*)
graphite	*R*_int_ = 0.042
φ and ω scans	θ_max_ = 28.4°, θ_min_ = 2.3°
Absorption correction: multi-scan (*SADABS*; Sheldrick, 1996)	*h* = −12→13
*T*_min_ = 0.897, *T*_max_ = 0.955	*k* = −18→17
10410 measured reflections	*l* = −11→11

### Refinement

**Table:**

Refinement on *F*^2^	Primary atom site location: structure-invariant direct methods
Least-squares matrix: full	Secondary atom site location: difference Fourier map
*R*[*F*^2^ > 2σ(*F*^2^)] = 0.034	Hydrogen site location: inferred from neighbouring sites
*wR*(*F*^2^) = 0.091	H-atom parameters constrained
*S* = 1.07	*w* = 1/[σ^2^(*F*_o_^2^) + (0.0323*P*)^2^ + 0.5573*P*] where *P* = (*F*_o_^2^ + 2*F*_c_^2^)/3
2657 reflections	(Δ/σ)_max_ = 0.001
146 parameters	Δρ_max_ = 0.30 e Å^−^^3^
0 restraints	Δρ_min_ = −0.26 e Å^−^^3^

### Special details

**Table:**

Geometry. All e.s.d.'s (except the e.s.d. in the dihedral angle between two l.s. planes) are estimated using the full covariance matrix. The cell e.s.d.'s are taken into account individually in the estimation of e.s.d.'s in distances, angles and torsion angles; correlations between e.s.d.'s in cell parameters are only used when they are defined by crystal symmetry. An approximate (isotropic) treatment of cell e.s.d.'s is used for estimating e.s.d.'s involving l.s. planes.
Refinement. Refinement of *F*^2^ against ALL reflections. The weighted *R*-factor *wR* and goodness of fit *S* are based on *F*^2^, conventional *R*-factors *R* are based on *F*, with *F* set to zero for negative *F*^2^. The threshold expression of *F*^2^ > σ(*F*^2^) is used only for calculating *R*-factors(gt) *etc*. and is not relevant to the choice of reflections for refinement. *R*-factors based on *F*^2^ are statistically about twice as large as those based on *F*, and *R*- factors based on ALL data will be even larger.

### Fractional atomic coordinates and isotropic or equivalent isotropic displacement parameters (Å^2^)

**Table:**

	*x*	*y*	*z*	*U*_iso_*/*U*_eq_	
Cl1	0.11909 (5)	0.35378 (4)	0.82090 (6)	0.03367 (14)	
Cl2	0.15267 (6)	0.72198 (3)	0.62638 (7)	0.03884 (15)	
O1	0.52503 (15)	0.37232 (9)	0.02888 (17)	0.0290 (3)	
N1	0.42007 (16)	0.47759 (11)	0.15589 (18)	0.0240 (3)	
H1	0.4313	0.5229	0.0888	0.029*	
N2	0.35554 (16)	0.50487 (11)	0.26436 (18)	0.0239 (3)	
C1	0.47026 (19)	0.38769 (13)	0.1379 (2)	0.0234 (4)	
C2	0.45389 (19)	0.31751 (13)	0.2541 (2)	0.0258 (4)	
H2	0.4893	0.2536	0.2543	0.031*	
C3	0.38829 (19)	0.34224 (13)	0.3631 (2)	0.0246 (4)	
H3	0.3760	0.2954	0.4387	0.029*	
C4	0.33735 (18)	0.43848 (12)	0.3652 (2)	0.0214 (3)	
C5	0.26737 (18)	0.47099 (12)	0.4848 (2)	0.0218 (3)	
C6	0.22820 (18)	0.40586 (13)	0.5865 (2)	0.0239 (4)	
H6	0.2436	0.3386	0.5781	0.029*	
C7	0.16682 (18)	0.43931 (13)	0.6996 (2)	0.0243 (4)	
C8	0.13991 (18)	0.53629 (13)	0.7192 (2)	0.0241 (4)	
C9	0.17893 (19)	0.59892 (13)	0.6130 (2)	0.0254 (4)	
C10	0.23982 (18)	0.56857 (13)	0.4983 (2)	0.0238 (4)	
H10	0.2630	0.6143	0.4284	0.029*	
C11	0.0760 (2)	0.57125 (15)	0.8451 (2)	0.0332 (4)	
H11C	0.1557	0.5816	0.9551	0.050*	
H11A	0.0072	0.5229	0.8573	0.050*	
H11B	0.0235	0.6321	0.8053	0.050*	

### Atomic displacement parameters (Å^2^)

**Table:**

	*U*^11^	*U*^22^	*U*^33^	*U*^12^	*U*^13^	*U*^23^
Cl1	0.0373 (3)	0.0338 (3)	0.0379 (3)	−0.00164 (19)	0.0233 (2)	0.0084 (2)
Cl2	0.0529 (3)	0.0248 (2)	0.0479 (3)	0.0092 (2)	0.0292 (3)	0.0019 (2)
O1	0.0396 (7)	0.0248 (7)	0.0304 (7)	0.0001 (5)	0.0220 (6)	−0.0012 (5)
N1	0.0319 (8)	0.0224 (7)	0.0227 (8)	0.0001 (6)	0.0157 (6)	0.0023 (6)
N2	0.0283 (7)	0.0235 (7)	0.0240 (8)	0.0003 (6)	0.0147 (6)	0.0006 (6)
C1	0.0247 (8)	0.0223 (8)	0.0242 (9)	−0.0025 (6)	0.0101 (7)	−0.0022 (7)
C2	0.0304 (9)	0.0192 (8)	0.0292 (10)	−0.0006 (7)	0.0127 (8)	0.0000 (7)
C3	0.0286 (9)	0.0219 (9)	0.0250 (9)	−0.0022 (7)	0.0120 (7)	0.0017 (7)
C4	0.0222 (8)	0.0216 (8)	0.0210 (8)	−0.0020 (6)	0.0087 (7)	−0.0003 (7)
C5	0.0202 (8)	0.0243 (9)	0.0203 (8)	−0.0014 (6)	0.0068 (6)	0.0003 (7)
C6	0.0239 (8)	0.0230 (9)	0.0265 (9)	−0.0003 (7)	0.0112 (7)	0.0009 (7)
C7	0.0226 (8)	0.0285 (9)	0.0231 (9)	−0.0019 (7)	0.0099 (7)	0.0037 (7)
C8	0.0197 (8)	0.0303 (9)	0.0220 (9)	0.0008 (7)	0.0072 (7)	−0.0012 (7)
C9	0.0261 (9)	0.0233 (9)	0.0266 (9)	0.0038 (7)	0.0096 (7)	0.0002 (7)
C10	0.0241 (8)	0.0244 (9)	0.0246 (9)	0.0010 (7)	0.0109 (7)	0.0037 (7)
C11	0.0374 (10)	0.0373 (11)	0.0296 (10)	0.0043 (8)	0.0178 (8)	−0.0001 (9)

### Geometric parameters (Å, °)

**Table:**

Cl1—C7	1.7404 (18)	C5—C10	1.391 (3)
Cl2—C9	1.733 (2)	C5—C6	1.395 (2)
O1—C1	1.244 (2)	C6—C7	1.384 (2)
N1—N2	1.345 (2)	C6—H6	0.9500
N1—C1	1.367 (2)	C7—C8	1.390 (3)
N1—H1	0.8800	C8—C9	1.400 (3)
N2—C4	1.311 (2)	C8—C11	1.500 (2)
C1—C2	1.435 (3)	C9—C10	1.378 (2)
C2—C3	1.347 (3)	C10—H10	0.9500
C2—H2	0.9500	C11—H11C	0.9800
C3—C4	1.425 (2)	C11—H11A	0.9800
C3—H3	0.9500	C11—H11B	0.9800
C4—C5	1.485 (2)		
			
N2—N1—C1	127.50 (15)	C7—C6—H6	120.1
N2—N1—H1	116.2	C5—C6—H6	120.1
C1—N1—H1	116.2	C6—C7—C8	123.71 (16)
C4—N2—N1	117.27 (15)	C6—C7—Cl1	117.35 (14)
O1—C1—N1	120.54 (16)	C8—C7—Cl1	118.93 (14)
O1—C1—C2	125.54 (17)	C7—C8—C9	114.45 (16)
N1—C1—C2	113.92 (16)	C7—C8—C11	122.88 (17)
C3—C2—C1	120.00 (17)	C9—C8—C11	122.67 (17)
C3—C2—H2	120.0	C10—C9—C8	123.67 (17)
C1—C2—H2	120.0	C10—C9—Cl2	117.27 (14)
C2—C3—C4	120.11 (16)	C8—C9—Cl2	119.06 (14)
C2—C3—H3	119.9	C9—C10—C5	120.05 (16)
C4—C3—H3	119.9	C9—C10—H10	120.0
N2—C4—C3	121.12 (16)	C5—C10—H10	120.0
N2—C4—C5	116.03 (15)	C8—C11—H11C	109.5
C3—C4—C5	122.79 (15)	C8—C11—H11A	109.5
C10—C5—C6	118.21 (16)	H11C—C11—H11A	109.5
C10—C5—C4	120.12 (15)	C8—C11—H11B	109.5
C6—C5—C4	121.67 (16)	H11C—C11—H11B	109.5
C7—C6—C5	119.90 (17)	H11A—C11—H11B	109.5
			
C1—N1—N2—C4	−0.2 (3)	C4—C5—C6—C7	178.27 (15)
N2—N1—C1—O1	178.37 (16)	C5—C6—C7—C8	0.3 (3)
N2—N1—C1—C2	−2.0 (3)	C5—C6—C7—Cl1	179.41 (13)
O1—C1—C2—C3	−177.86 (17)	C6—C7—C8—C9	0.6 (3)
N1—C1—C2—C3	2.6 (2)	Cl1—C7—C8—C9	−178.51 (13)
C1—C2—C3—C4	−1.1 (3)	C6—C7—C8—C11	−178.75 (17)
N1—N2—C4—C3	1.9 (2)	Cl1—C7—C8—C11	2.2 (2)
N1—N2—C4—C5	179.33 (14)	C7—C8—C9—C10	−0.3 (3)
C2—C3—C4—N2	−1.3 (3)	C11—C8—C9—C10	178.98 (17)
C2—C3—C4—C5	−178.51 (16)	C7—C8—C9—Cl2	−179.37 (13)
N2—C4—C5—C10	−6.9 (2)	C11—C8—C9—Cl2	−0.1 (2)
C3—C4—C5—C10	170.44 (16)	C8—C9—C10—C5	−0.8 (3)
N2—C4—C5—C6	173.38 (16)	Cl2—C9—C10—C5	178.26 (13)
C3—C4—C5—C6	−9.3 (3)	C6—C5—C10—C9	1.7 (3)
C10—C5—C6—C7	−1.5 (3)	C4—C5—C10—C9	−178.06 (15)

### Hydrogen-bond geometry (Å, °)

**Table:**

*D*—H···*A*	*D*—H	H···*A*	*D*···*A*	*D*—H···*A*
N1—H1···O1^i^	0.88	1.90	2.771 (2)	172

Symmetry codes: (i) −*x*+1, −*y*+1, −*z*.
